# Case Report: Successful Response to Intravenous Immunoglobulin and Steroid Pulses in a Renal Transplant Recipient With Severe Covid-19 Disease and Associated Acute Allograft Failure

**DOI:** 10.3389/fimmu.2021.671013

**Published:** 2021-05-11

**Authors:** Pedro Rosa-Guerrero, Antonio Trujillo-Aguilera, Juan Molina, Ana Navas, Cristina López-Martín, Aurora Jurado, Alberto Rodríguez-Benot, Álvaro Torres-De-Rueda

**Affiliations:** ^1^ Maimonides Biomedical Research Institute of Cordoba (IMIBIC), Reina Sofia University Hospital, University of Cordoba, Cordoba, Spain; ^2^ Department of Nephrology, Reina Sofia University Hospital, Cordoba, Spain; ^3^ Department of Immunology and Allergy, Reina Sofia University Hospital, Cordoba, Spain; ^4^ Department of Intensive Care, Reina Sofia University Hospital, Cordoba, Spain; ^5^ Asociación Medicina e Investigación (A.M.I.), Cordoba, Spain

**Keywords:** Covid-19, SARS-CoV-2, kidney transplantation, intravenous immunoglobulin, steroids, cytokine storm, case report

## Abstract

The impact of Covid-19 pneumonia caused by SARS-CoV-2 on transplanted populations under chronic immunosuppression seems to be greater than in normal population. Clinical management of the disease, particularly in those patients worsening after a cytokine storm, with or without allograft impairment and using available therapeutic approaches in the absence of specific drugs to fight against the virus, involves a major challenge for physicians. We herein provide evidence of the usefulness of high-dose intravenous immunoglobulin (IVIG) combined with steroid pulses to successfully treat a case of Covid-19 pneumonia in a single-kidney transplanted patient with mechanical ventilation and hemodialysis requirements in the setting of a cytokine storm. A rapid decrease in the serum level of inflammatory cytokines, particularly IL-6, IL-8, TNF-α, MCP-1 and IL-10, as well as of acute-phase reactants such as ferritin, D-dimer and C-reactive protein was observed after the IVIG infusion and methylprednisolone bolus administration with a parallel clinical improvement and progressive allograft function recovery, allowing the patient’s final discharge 40 days after the treatment onset. The immunomodulatory effect of IVIG together with the anti-inflammatory and immunosuppressive potential of steroids could be an alternative strategy to treat severe cases of Covid-19 pneumonia associated with an uncontrolled inflammatory response in transplanted populations.

## Introduction

Although the majority of Covid-19 disease clinical manifestations range from asymptomatic to mild respiratory infections, an important number of patients undergo symptomatic or severe pneumonia with acute respiratory distress syndrome (ARDS) and concomitant life-threatening complications (1). Solid-organ transplanted recipients comprise a particularly vulnerable group given their increased susceptibility to infections as a consequence of chronic immunosuppression and coexisting conditions (2). In such a population, frontline adopted strategies for managing the infection, beside conventional care practice, are mainly based on the adjustment of baseline immunosuppressive regimens with the purpose of enhancing the host-response against the virus (3). Unfortunately, the time-course of Covid-19 disease is known to be highly erratic and initial therapeutic efforts are not often enough to avoid a poor progression in susceptible individuals. Importantly, successful treatments for patients undergoing a critical situation secondary to infection are urgently needed.

One of the most relevant findings amongst patients with more severe forms of the disease is the presence of high levels of circulating cytokines and subsequent acute-phase reactants (4), suggesting that an innate-immunological dysregulation characterized by a massive cytokine release (so-called *cytokine-storm*) may be associated with a worsening of the clinical syndrome involving multiple organ failure and higher rates of fatal outcomes. Accordingly, a reasonable therapeutic approach should be addressed to limit the collateral host-tissues damage promoted by the hyperinflammatory state, beyond the use of drugs specifically targeting the virus or advanced life-support measures.

## Case Description

**Figure 1:**
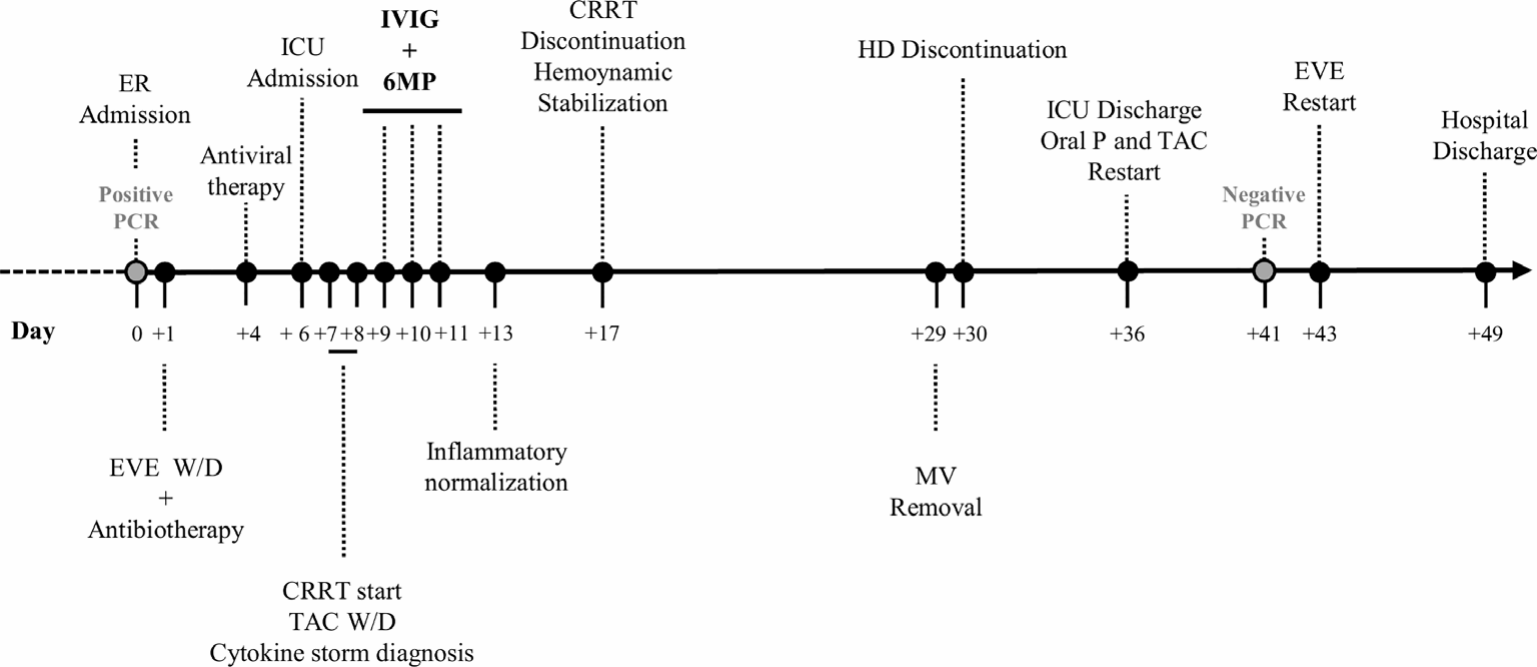
Timeline of hospitalization. CRRT, continuous renal replacement therapy; EVE, everolimus; ER, emergency room; HD, hemodialysis; ICU, intensive care unit; IVIG, intravenous immunoglobulin; MV, mechanical ventilation; oral P, oral prednisone; TAC, tacrolimus; W/D, withdrawal; 6MP, 6-methylprednisolone.

During the course of the first wave of the pandemic, a 54-year-old man with end-stage renal disease of unknown etiology, recipient of a renal allograft in 2015 under combined maintenance immunosuppression with prednisone, tacrolimus (TAC) and everolimus (EVE), baseline serum creatinine of 1.2 mg/dL and estimated glomerular filtration rate of 67 mL/min, was admitted to Emergency Room on 19^th^ March with a 5-day history of fever and dyspnea unresponsive to paracetamol and levofloxacin intake (**Figure 1**). On examination, chest-X-ray revealed multiple bilateral patchy ground-glass opacities. The nasopharyngeal SARS-CoV-2 RNA-PCR was positive and the patient was diagnosed with Covid-19 associated bilateral pneumonia. His past medical history was remarkable for left-native kidney radical nephrectomy in 2006 after a focal clear-cell adenocarcinoma diagnosis; obstructive sleep apnea syndrome managed with continuous positive airway pressure and long-standing hypertension controlled with doxazosin, enalapril and manidipine.

**Figure 2:**
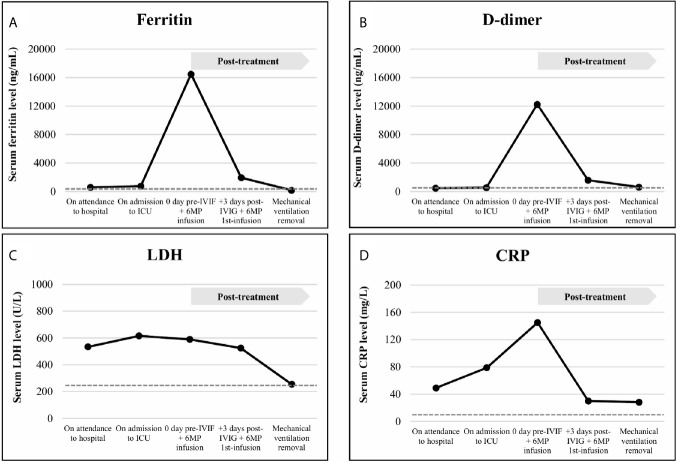
Acute-phase reactants levels, including ferritin **(A)**, D-dimer **(B)**, lactate dehydrogenase (LDH, **C**) and C-reactive protein (CRP, **D**), analyzed in serum samples of the patient on attendance to hospital date, on admission to Intensive Care Unit (ICU) date, on the day of the intravenous immunoglobulin (IVIG) plus 6-methylprednisolone (6MP) infusion, early (3 days) after the IVIG plus 6MP infusion and on mechanical ventilation removal. Normal range laboratory values are displayed in broken lines. The treatment administration is indicated in a grey box. Ferritin was assessed from serum samples by sandwich immunoassay using direct chemiluminescence technology (Atellica IM Ferritin -Fer-, ref. 10995568) on an Atellica^®^ IM Analyzer (Siemens Healthineers Diagnostics Inc.). D-dimer was determined from citrated plasma by automated latex enhanced immunoassay (ref. 0020008500, Instrumentation Laboratory, Werfen) on an ACL TOP 700 LAS (Werfen). LDH was quantified from serum samples according to the proportional increase in the absorbance at 340/310 nm of NADH (Atellica CH Lactate Dehydrogenase L-P -LDPL-, ref. 11097594) on an Atellica^®^ CH Analyzer (Siemens Healthineers Diagnostics Inc.). CRP was measured from serum samples by latex enhanced immunoturbidimetry assay (Atellica CH Wide Range C-Reactive Protein -wrCRP-, ref. 11097645) on an Atellica^®^ CH Analyzer (Siemens Healthineers Diagnostics Inc.). All the automatic assays were performed according to manufacturer instructions.

**Figure 3:**
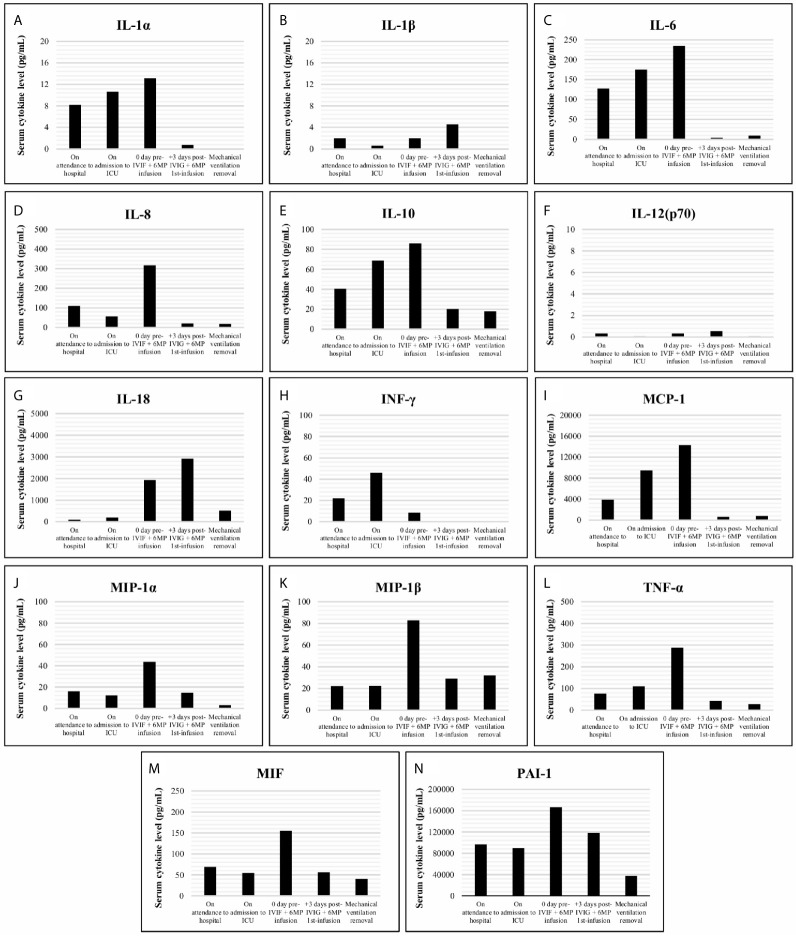
Cytokine levels (pg/mL) including IL-1α, IL-1β, IL-6, IL-8, IL-10, IL-12(p70), IL-18, INF-γ, MCP-1, MIP-1α, MIP-1β, TNF-α, MIF and PAI-1 **(A–N)**, analyzed in serum samples of the patient obtained on attendance to hospital date, on admission to Intensive Care Unit (ICU) date, on the day of the intravenous immunoglobulin (IVIG) plus 6-methylprednisolone (6MP) infusion, early (3 days) after the IVIG plus 6MP infusion and on mechanical ventilation removal. Cytokine measurements were retrospectively performed on -80 °C preserved samples, except for IL-6 (prospectively analyzed), using customized Milliplex^®^ MAP kits (Panel **A**: IL-1α, IL-1β, IL-6, IL-8, IL-10, IL-12(p70), IL-18, INF-γ, MCP-1, MIP-1α, MIP-1β and TNF-α, ref. HCYTA-60K; Panel **B**: MIF and PAI-1, ref. HSP1MAG-63K; Merk-Millipore) according to manufacturer instructions on a Luminex platform with xPONENT vs. 4.2 as acquisition and analysis software.

On admission, piperacillin-tazobactam 4/0.5 g/8h plus azithromycin 250 mg od, and hydroxychloroquine 200 mg od were started, whilst EVE was discontinued and TAC dose was lowered. On day +4 after admission, lopinavir/ritonavir 400/100 mg/12h was added. On day +6, the patient evolved into a progressive respiratory failure, requiring mechanical ventilation in the intensive care unit (ICU). At that point, all immunosuppression was discontinued, only maintaining 6-methylprednisolone (6MP) 40 mg IV/24h. A total of 4 doses of lopinavir/ritonavir and 1 dose of β-Interferon (250 µg on day +7) were given. In the first 48h at ICU, the patient’s general condition worsened, presenting oliguric acute kidney failure with an active urine sediment, which was treated with continuous renal replacement therapy (CRRT). The global clinical course linked to radiologic deterioration and biochemical findings (ferritin > 16500 ng/mL; D-dimer 12255 ng/mL; c-reactive protein 145.6 mg/L; lactate dehydrogenase 590 U/L; IL-6 234.7 pg/mL; **Figures 2**, **3**) became highly suggestive of an unbalanced systemic inflammatory response in the context of a *cytokine-storm*. In this setting, a three-day consecutive course of high-dose intravenous immunoglobulin (IVIG) 65 g/day (an accumulated 2.2 g/kg) and 125 mg/day of 6MP were administered (days +9, +10 and +11 since admission; **Figure 1**).

This cycle was followed by a rapid and significant improvement in the pro-inflammatory profile (ferritin 1949.1 ng/mL; D-dimer 1597 ng/mL; c-reactive protein 29.9 mg/L; lactate dehydrogenase 525 U/L; IL-6 4.1 pg/mL; **Figures 2**, **3**) with hemodynamic stabilization and discontinuation of CRRT six days after IVIG plus corticosteroid pulses completion (having received a total course of 9 days of CRRT). The patient continued under alternative-day renal replacement therapy with hemodialysis for another 13 days and was extubated after 23 days on mechanical ventilation, remaining up-to a total of 30 days in the ICU. Diuresis and renal function progressively recovered, being restarted on oral prednisone and TAC on +36 day after admission, followed by EVE one week later. SARS-CoV-2 RNA-PCR became negative on day +41. The patient was discharged after 49 hospital days (**Figure 1**) with a serum creatinine of 2.3 mg/dL, 1200 mg/L proteinuria and resumed regular outpatient clinic reviews. The anti-HLA antibody panel determined at the time of discharge remained negative, suggesting the absence of humoral alloresponse. Current general condition remains satisfactory without clinical findings of allograft impairment (last follow-up serum creatinine was 1.65 mg/dL) or respiratory sequels.

## Discussion

Herein we present a case of a SARS-CoV-2 infected single-kidney transplanted patient with ARDS and acute kidney-allograft failure under a depleted immunosuppressant regimen with the hallmark of associated cytokine-storm, who was treated with high doses of IVIG and steroid pulses in an attempt mainly to reduce the excessive inflammatory response but also to prevent eventual rejection damage, supported by their well-known immunomodulatory and immunosuppressive effects. The compassionate use of tocilizumab, an IL-6 receptor antagonist potentially able to mitigate the cytokine release syndrome induced by SARS-CoV-2 (5), although being considered, was finally dismissed due to a reasonable clinical suspicion of concomitant bacterial infection, which nevertheless was later discarded. Interestingly, a dramatic clearance of the inflammatory cytokine network, as well as acute-phase reactants, was observed in a short time-frame once the treatment was administered as depicted in **Figures 2**, **3** respectively, with a parallel clinical improvement of the patient, including progressive hemodynamic stabilization and gradual recovery of renal-allograft function with neither evidence of clinical rejection nor development of donor-specific anti-HLA antibodies.

Beyond the cytopathic organ injury caused by SARS-CoV-2 targeting ACE-2 expressing host-tissues (6), the clinical severity was in this case exacerbated by the extensive inflammatory damage induced by an anomalous late release of cytokines in response to the unresolved infection (7). A progressive rise since admission of IL-6, IL-8, IL-10, and TNF-α as well as other key mediators (e.g., MCP-1, PAI-I or MIF) largely related with the development of sepsis (8, 9), was found. High levels of circulating inflammatory cytokines exert a pernicious effect on endothelium, causing the disruption of its function as a barrier with the subsequent fluid leakage to intercellular spaces and the imbalance of homeostatic equilibrium into a procoagulant and antifibrinolytic state, resulting in microvascular thrombosis and organ ischemia. Moreover, this hyperinflammatory environment promotes oxidative stress damage through an increased production of reactive oxygen species (10). Altogether, these systemic alterations could drive the progression of the infection towards ARDS and acute renal-allograft failure, major complications associated with poorer outcome in Covid-19 disease (11, 12).

Inflammatory profile normalization, before an irreversible multi-organ impairment, is expected to be critical for recovery. A reduction of plasma IL-6 level has been already associated with favorable prognosis during the septic insult, whereas the persistent overproduction of IL-10, which should act as a key negative regulator controlling the magnitude of the response, paradoxically seems to be the main fatality predictor by inducing a robust immunosuppressant state (8). From this perspective, the positive response here described was most probably linked to the rapid decrease of both pro- and anti-inflammatory cytokines mediated by the treatment. Despite the global decrease, some particular behaviors were detected, as in the case of the pro-inflammatory IL-18, which transiently increased after IVIG plus 6MP infusion (**Figure 3G**). This finding could partially be explained by the lack of negative feedback as a result of the circulating INF-γ low level at that point (**Figure 3H**), suppressing the expression of the high affinity IL-18 binding protein, a physiological IL-18 inhibitor (13). In turn, the reduction of INF-γ, whose main source of production are activated T- and NK-cells, during the first 48h in ICU before treatment, could be secondary to the functional exhaustion of these lymphocytes (14) after a prolonged unsuccessful response against SARS-CoV-2, being this fact followed by or overlapped the disease progression.

It has been suggested that the mechanism by which IVIG leads to immunomodulation depends on both the immunoglobulin Fc and F(ab´)2 regions. On one hand, high-doses of IVIG can saturate Fcγ-receptors performing an inhibitory role on innate-immune cells, decreasing their migration capacity and responsiveness to soluble mediators and also can sequester circulating complement molecules (15). On the other hand, it can neutralize inflammatory cytokines and apoptosis-inducing molecules by F(ab´) regions (15). The anti-inflammatory and immunosuppressive effects of glucocorticoids are mostly mediated through their receptor, an almost ubiquitously expressed transcription factor which, upon ligand binding, translocates towards the nucleus leading major changes in inflammatory gene expression programs, typically due to its physical interaction with specific DNA sequences as well as with transcription modulators such as NF-κβ or AP-1. Particularly, on monocytes-macrophages, whose dysfunction plays a pivotal role in the physiopathology of severe inflammation, corticoids inhibit or down-regulate the expression of several pro-inflammatory cytokines, limiting an overwhelming response (16). Regarding adaptive cellular immunity, IVIG and corticoids may alter the balance of T-cell subpopulations to favor an anti-inflammatory response by promoting the expansion and enhancement of CD4^+^FoxP3^+^ regulatory T-cells and by inhibiting the proliferation and activity of effector T-helper 1 cells (17, 18).

Further than being one of the mainstays to prevent rejection since the inception of solid-organ transplantation, corticoids have been widely used in managing inflammatory and autoimmune diseases, even showing clinical benefits to treat disseminated intravascular coagulation, sepsis or ARDS (19). In turn, IVIG is implemented in a broad spectrum of disorders, including some involving an uncontrolled immune response such as Kawasaki disease or toxic shock syndrome, which share similarities with severe Covid-19 forms (20). Despite both drugs could offer a rationale for its use in Covid-19 disease evolving towards a severe systemic inflammatory response, only the use of corticoids has been recommended when supplemental oxygen was required (21). In contrast, there is not an agreement about the feasible usefulness of IVIG to improve clinical outcomes in SARS-CoV-2 infected patients, notwithstanding a few publications support its effectiveness alone or combined with corticoids in more severe cases (22–24).

In conclusion, together with previous reports, the study of this case evinces that IVIG at immunomodulatory doses in combination with steroids could be an alternative successful strategy to control the cytokine-storm triggered by SARS-CoV-2 infection, improving the course of the disease and offering protection against immunological rejection in transplanted patients off their maintenance immunosuppression regimes. Since this is a single-case, more studies evaluating the effectiveness of this therapy would be necessary to extrapolate it to similar scenarios. Future studies designed to explore whether the administration of IVIG-6MP at early stages of the disease may provide a prophylactic effect by blocking the Covid-19 progression towards an hyperinflammatory syndrome would merit consideration, especially in view of the current lack of efficient treatments against the virus and the delicate equilibrium within the host immunity status required for both overcoming the infection and avoiding allograft rejection.

## Patient Perspective

Being a renal transplant patient taking immunosuppressive medication, I became very worried for my life when I was notified to have Covid-19 disease, but also for losing my kidney allograft. During my first days at the hospital, I rapidly worsened and was therefore transferred to ICU under artificial breathing. Of that horrible period, I only vaguely remember having nightmares. After regaining consciousness, I started a slow recovery, during which I often felt depressed and anxious, but I finally could leave the hospital off dialysis and with my kidney improving day by day. I am very thankful to the cooperative and multidisciplinary work that experts in different areas of knowledge performed and the treatment and the personal attention I received, and very especially to my daughter who, being a nurse in training, stayed long days of room isolation looking after me and working towards my recovery.

## Data Availability Statement

The data analyzed in this study is subject to the following licenses/restrictions: Patient’s clinical record. Requests to access these datasets should be directed to atorresnefro@yahoo.es.

## Ethics Statement

Ethical review and approval was not required for the study on human participants in accordance with the local legislation and institutional requirements. The patients/participants provided their written informed consent to participate in this study.

## Author Contributions

PRG, ATR, and CLM participated in the diagnosis and treatment of the patient. PRG and ATA collected clinical data. JM, AN, and ATA conducted cytokine measurements. PRG, ATA, JM, AN, and ATR wrote the draft. AJ and ARB reviewed the final version and made significant conceptual contributions to the manuscript. All authors contributed to the article and approved the submitted version.

## Funding

The publishing of this work was financed by Asociación Medicina e Investigación (A.M.I.).

## Conflict of Interest

The authors declare that the research was conducted in the absence of any commercial or financial relationships that could be construed as a potential conflict of interest.

## Abbreviations

ARDS, acute respiratory distress syndrome; CRRT, continuous renal replacement therapy; EVE, everolimus; ICU, intensive care unit; IVIG, intravenous immunoglobulin; TAC, tacrolimus; 6MP, 6-methylprednisolone.
